# Empirical validation of statistical parametric mapping for group imaging of fast neural activity using electrical impedance tomography

**DOI:** 10.1088/0967-3334/37/6/951

**Published:** 2016-05-20

**Authors:** B Packham, G Barnes, G Sato dos Santos, K Aristovich, O Gilad, A Ghosh, T Oh, D Holder

**Affiliations:** 1 Department of Medical Physics & Bioengineering, University College London, UK; 2 Wellcome Trust Centre for Neuroimaging, Institute of Neurology, University College London, UK; b.packham@ucl.ac.uk

**Keywords:** EIT, SPM, permutation testing, neuroimaging, functional brain imaging

## Abstract

Electrical impedance tomography (EIT) allows for the reconstruction of internal conductivity from surface measurements. A change in conductivity occurs as ion channels open during neural activity, making EIT a potential tool for functional brain imaging. EIT images can have  >10 000 voxels, which means statistical analysis of such images presents a substantial multiple testing problem. One way to optimally correct for these issues and still maintain the flexibility of complicated experimental designs is to use random field theory. This parametric method estimates the distribution of peaks one would expect by chance in a smooth random field of a given size. Random field theory has been used in several other neuroimaging techniques but never validated for EIT images of fast neural activity, such validation can be achieved using non-parametric techniques. Both parametric and non-parametric techniques were used to analyze a set of 22 images collected from 8 rats. Significant group activations were detected using both techniques (corrected *p*  <  0.05). Both parametric and non-parametric analyses yielded similar results, although the latter was less conservative. These results demonstrate the first statistical analysis of such an image set and indicate that such an analysis is an approach for EIT images of neural activity.

## 1. Introduction

Electrical impedance tomography (EIT) is an emerging medical imaging technique which allows for the reconstruction of the distribution of conductivity, or changes in this, from surface measurements. This is achieved by the application of a constant current to a series of surface electrodes, with simultaneous recording of the resulting voltages. As the applied current is constant, changes in the measured voltage can be used to determine changes in the internal conductivity (Bayford 2006). Ideally, one independent measurement is needed for each voxel in the reconstructed image. However, while images typically contain  >10 000 voxels, it is not usually practical to have more than 30–60 electrodes. This yields up to one thousand or so independent measurements, so that the inverse solution is under-determined. As a result, the spatial resolution is typically about 5–10% of the image diameter, and so is less than modalities such as MRI. Nonetheless, EIT has the advantages of a high temporal resolution (<1 ms), no ionizing radiation, low cost and portability (Lionheart 2004). Consequently, EIT has been proposed for multiple applications in biomedicine, including imaging of pulmonary perfusion (Nguyen *et al* 2012), gastric emptying (Smallwood *et al* 1994), breast (Zou and Guo 2003) and prostate malignancy (Borsic *et al* 2010) and acute cerebral stroke (Malone *et al* 2014). It is currently in clinical use for imaging lung inflation as a means of monitoring invasive ventilation (Frerichs 2000, Luecke *et al* 2012).

### 1.1. EIT of neural activity

Among the proposed applications of EIT is its use for the imaging of neural activity and associated haemodynamic changes (Tidswell *et al* 2001). Impedance has been shown to change during neural activity in modeling studies (Liston *et al* 2012), peripheral nerve (Cole and Curtis 1939, Gilad *et al* 2009), and cerebral cortical tissue (Adey *et al* 1962, Klivington and Galambos 1968, Oh *et al* 2011). This impedance change is caused by the opening of ion channels during action potentials and post-synaptic potentials. In peripheral nerve, the changes are up to  −1% (Gilad *et al* 2009, Oh *et al* 2011); however, the changes in the brain, recorded with scalp or subdural electrodes, are diminished by two or more orders of magnitude. This is due to volume conduction and the diversion of applied current, by the skull, so that a large proportion does not pass into the brain. Recently, the UCL EIT research group has produced tomographic images of fast neural activity, using EIT (Gilad *et al* 2010, Oh *et al* 2011, Aristovich *et al* 2016). However, the statistical analysis of this work presents a considerable multiple testing problem, due to the nature of EIT image reconstruction.

Commonly, in EIT image reconstruction a ‘sensitivity matrix’ is employed; a linear approximation is used to relate a map of small conductivity changes, $\delta \sigma $, within a finite-element method (FEM) mesh with respect to a baseline conductivity distribution, ${{\sigma}_{0}}$, to changes in boundary voltages, $\delta \mathbf{v}$:

\begin{eqnarray*}\delta \mathbf{v}=A\delta \boldsymbol{\sigma },\end{eqnarray*}

where $A$ is the sensitivity matrix, an *m*-by-*n* matrix, where *m* is the size of the collected data, typically  <1000, and *n* the size of the number of tetrahedra in the FEM mesh, typically  >10 000.

This formulation is referred to as the forward problem, with the pseudoinversion of $A$ allowing for calculation of the conductivity from the measured voltage changes. As the problem is underdetermined and the solution is ill-posed, the pseudo-inverse is performed with regularization of $A$ (Lionheart *et al* 2005). A critical component in minimizing errors in the forward solution is ensuring a sufficient number of elements are present in the FEM mesh, which can be greater than several million elements or voxels. This has previously been explored by assessing mesh convergence, which relates to refining the element size near the electrodes until the convergence error reaches a required precision (Aristovich *et al* 2014).

In this study, we are concerned with the construction of statistical parametric maps, which involves a mass-univariate approach: the same General Linear Model (GLM) is applied at each voxel. This gives rise to images consisting of thousands of statistical tests—one for each voxel. These numerous statistical tests give rise to an inflated false positive rate. For example at a test wise significance level of 0.05 and 10 000 independent test; then by definition even if there is no experimental effect one could expect up to 500 significant findings.

### 1.2. Analysis of EIT images

To date, the only application of mass-univariate analysis to EIT images has been first-level analysis of haemodynamic responses, whereas the image set presented here relates directly to fast neural activity. Zhang *et al* undertook first-level analysis on simulated EIT images of haemodynamic responses, both without and with added noise. The employment of SPM allowed for the localization of significant changes whose center were  <8.5% displaced from the simulated perturbation’s center (Zhang *et al* 2005). Similarly, Yerworth *et al* undertook first-level analysis on simulated, tank phantom, and human data of visual evoked potentials secondary to checkerboard stimulation in 14 healthy adults. In both the simulation and tank experiments, averaged EIT images were deemed comparable to SPMs (statistical parametric maps) and resulted in a significant area in the occipital region of the head (*p*  <  0.001). However, the SPM results for human data, as with the EIT images, were seemingly artefactual, with significant results also in the anterior and lateral aspects of the brain (Yerworth *et al* 2007).

Given the minimal application of SPM to EIT images, consideration must be given to the unique aspects of EIT as compared to techniques such as fMRI; EIT suffers from a poor spatial resolution and comparably large point spread function, which may result in persistent artifacts that would be identified as significant activations by SPM. In addition to this, SPM has never been applied to EIT imaging of fast neural activity or EIT imaging undertaken with a planar electrode array, both of which might uniquely affect interpretation of SPMs; sensitivity drops exponentially with increased distance from a planar array and can result in a larger increase in the point spread function in the *z*-axis (Mueller *et al* 1999, Kao *et al* 2006).

In this work, we compare three distinct methods for controlling family wise error rate (FWE) across a large number of EIT image voxels. These are Bonferroni correction, random field theory (RFT) and permutation testing, and are briefly outlined below. A more detailed background to RFT and permutation testing have been detailed numerous times previously (for example Friston *et al* (2006)).

#### 1.2.1. Bonferroni correction.

The simplest way to mitigate an inflated family wise error rate is to use a Bonferroni correction, which adjusts the significance level to account for the number of independent tests:

\begin{eqnarray*}\alpha ={{p}^{\text{FWE}}}/n,\end{eqnarray*}

where, *α* is the voxel level threshold value against which significance is determined, *n* is the number of tests and ${{p}^{\text{FWE}}}$ is the desired false positive rate for the whole volume. In this case, assuming a total image of 10 000 voxels, we would only accept individual voxels as significant if $p&lt;0.05/10\,000$.

#### 1.2.2. Random field theory.

The problem with Bonferroni correction is that, in most neuroimaging modalities, voxels are not independent, but in fact locally correlated. This is especially true in EIT where the underdetermined nature of the image and low spatial resolution result in substantial spatial correlation, which would make a Bonferroni correction thoroughly inappropriate. In order to take advantage of this local smoothness structure, one can employ techniques like random field theory, which effectively predicts how many peaks and troughs one might expect in a smooth image of, for example, *t*-statistics on random data (Worsley *et al* 1992, Kiebel *et al* 1999).

By knowing the smoothness and volume occupied by an image, we can make direct analytic predictions of what would be unusual (*p*  <  0.05) in a random field of this size. Using this family wise correction, under the null hypothesis, the resultant maps of significant *t*- or *F*-values are displayed as images (Worsley *et al* 1992). These tests can be applied to single subject experiment observations, referred to as first-level analysis. Alternatively, inferences can be made across data collected from multiple subjects, and so a population’s estimated responses tested for statistical significance, referred to as second-level analysis (Holmes and Friston 1998, Friston *et al* 2005).

The principle underpinning the family-wise correction in random field theory is that, because many voxels are spatially correlated, the number of independent tests that need correcting for is less than the number of voxels. This is addressed in random field theory by considering the number of resels and Euler characteristic of an image domain. Resels, or resolution elements, are spatially correlated groups of voxels and are estimated from the effective full width at half maximum (FWHM). While the FWHM is known for a smoothing kernel, if one was employed, the effective FWHM will often be different and dependent on location and so is estimated. This estimation is based upon the spatial derivative of the normalized least-squares residuals calculated in the estimation procedure for the GLM. Therefore, in principle, while EIT and other neuroimaging techniques have very different properties, such as differing spatial resolutions, so long as the error terms of the GLM meet certain assumptions, the correction can be calculated from the images’ properties (Friston *et al* 2006).

There are specific assumptions placed upon the error terms of the GLM, which include that the errors have a constant variance in each observation with a mean around zero, and that the errors are independent-they are non-autocorrelative across observations. The assumptions underpinning RFT are that the error fields in the data are a reasonable lattice approximation of an underlying random field with a multivariate Gaussian distribution and that the fields are continuous. These assumptions can be broken if the data is not smooth or if the GLM was incorrectly specified (Friston *et al* 1995, Kiebel and Holmes 2007, Worsley 2007).

#### 1.2.3. Permutation testing.

It would therefore be valuable to apply SPM to a cohort of EIT images of neural activity, but the validity of doing so might need to be tested as SPM and RFT require the fulfillment of certain assumptions. Validation of the use of RFT can be achieved through the use of nonparametric methods; such as permutation testing as implement in statistical non-parametric mapping (SnPM).

In permutation testing, the data is used to generate the probability distribution, rather than assuming a particular distribution (i.e. *t*-distribution). The main assumption behind permutation testing is that under the null hypothesis relabeling of the data will have no effect. For example, at a first-level analysis this might involve replacing ‘active’ with ‘passive’ trials or at a second level analysis switching the sign of the effect size (active-passive) on certain subjects. With this assumption in place, the data are reassigned to a new labeling, which for second-level analysis involves multiplication of data from each subject by either 1 or  −1. The rationale is that, under the null hypothesis (the change over subjects is zero), the multiplication of each subject’s effect size by either 1 or  −1 will have no effect. There are many possible permutations of relabeling and this allows one to build up a distribution of statistical images. The multiple-comparison problem is automatically dealt with by constructing a null-distribution based on the maximum values from each of these images (Nichols and Hayasaka 2003). However, permutations testing can be computationally demanding as the total number of possible permutations is 2^*N*^, where *N* is the number of relabelings. Often this many permutations would represent an unacceptable computational burden and so Monte Carlo testing is often employed, in which a random subsample, ${{N}^{\prime}}$, of all the possible relabellings is taken. While this reduces the power of the test (Nichols and Hayasaka 2003), as few as 1000 permutations in the subset can be sufficient to maintain power in Monte Carlo testing (Edgington 1969). The error that is introduced can be calculated as $2\sqrt{p(1-p)/{{N}^{\prime}}}$ (Jocekl 1986). Hence, while permutation testing lacks the assumptions of RFT it is still preferable to be able to employ RFT. First, permutation testing is computationally more demanding than RFT, as randomization of each voxel, or at least several thousand voxels, must be undertaken and secondly, expanding permutation testing to more complex designs is somewhat more complex than the RFT equivalents. For these reasons it is desirable to use RFT, but the validity of its use should ideally be examined first.

In this paper, we set out to compare parametric (random field theory) and non-parametric (permutation testing) approaches to controlling the false positive rate for group-level analysis of EIT images of fast neural activity. The purpose was to determine if SPM is a tenable approach to statistical analysis of EIT images of fast neural activity by using non-parametric methods as a gold standard against which to compare SPM results. The paper proceeds as follows. We first describe the collection and pre-processing of an EIT dataset based on somatosensory stimulation on a cohort of anesthetized rats. This gives us a set of 3D images per rat which evolve in time over a 40 ms time window. We then test for significant experimental effects, correcting for multiple comparisons throughout the volume either using parametric (RFT) or non-parametric (permutation) approaches. In addition, a Bonferroni correction approach was also employed. While it was expected from first principles and its use in analyzing other neuroimages that it would yield more conservative results than parametric (RFT) or non-parametric techniques its use was mainly for comparative reasons to offer a reference point for those unfamiliar with the use of SPM or SnPM. Indeed we did find that both parametric and non-parametric methods are much more sensitive than a Bonferroni correction, but also that parametric approaches are marginally more conservative than non-parametric approaches for these data.

## 2. Methods

### 2.1. Data cohort and collection

EIT images were reconstructed from data collected during somatosensory evoked cerebral activity in the anesthetized rat, using an epicortical planar electrode array, $7\times 5$ mm with 29 electrodes, each 0.6 mm in diameter. It was constructed of platinum foil on a silicone rubber backing. It was placed over exposed somatosensory cerebral cortex. Somatosensory stimulation was produced by 2 Hz electrical stimulation of the contralateral median nerve. Current for impedance recording was injected at 50 *μ*A and 225 Hz through a single electrode pair in the planar epidural electrode array for 70 s. During each current injection, the remaining 27 electrodes recorded voltage. This was repeated by switching the current injection pair, for 30 different electrode pairs, so that a total of approximately 900 four terminal traces were recorded. The first 10 s of each injection was discarded to remove switch related artifacts, yielding a 60 s segment with 120 stimulations. The average of the phase and antiphase was subsequently added or subtracted to yield the evoked potential or modulated impedance change, respectively. The impedance data was filtered with a bandwidth of 100–350 Hz to minimize EEG noise within the recordings, as EEG power is largely  <100 Hz; however, a 250 Hz bandwidth limited reliable temporal feature extraction to 8 ms (Oh *et al* 2011). Post-mortem recordings were also undertaken as a control.

The final data were 300 ms long, 50 ms pre-stimulus and 250 ms post-stimulus. The pre-stimulus data were used to calculate voltage differences. Some of the recorded voltages were rejected or not recorded if the noise in the pre-stimulus time was greater than 0.3 *μ*V or 0.01% of the boundary voltages, there was significant 50 Hz noise, or if there was a faulty electrode contact. After rejection, the noise was $0.18\pm 0.04$ *μ*V (mean  ±  standard deviation; range 0.10–0.24 *μ*V) across the 24 recordings in 8 rats, of which 2 recordings were controls (table 1). From this process, images were reconstructed with the real component of voltage differences taken at 21 time points, 0–40 ms every 2 ms. All data processing and image reconstruction was undertaken using MATLAB (The MathWorks, Inc., Natick, Massachusetts, USA). The methodology of the data acquisition and processing is described in Gilad *et al* (2010) and Oh *et al* (2011).

**Table 1.** Distribution of recordings across rats.

Rat number	Active recordings	Controls
1	4	
2	3	
3	3	
4	2	
5	3	
6	2	
7	1	2
8	4	

### 2.2. Image reconstruction and preprocessing

The voltage differences were reconstructed into conductivity difference images using a sensitivity matrix reconstruction algorithm (Bagshaw *et al* 2003). In solving the forward problem the conductivity of the brain was taken to be 0.3 Sm^−1^, homogeneous and isotopic. Calculations were performed using the UCL SuperSolver package, which is based on the EIDORS package (Adler and Lionheart 2006). A rat brain FEM mesh was used, which had 3 000 000 tetrahedra, with refinement over the region of electrode placement. Electrode array positions were determined post-hoc by analysis of the distribution of the topography of the EPs’ (appendix). Inversion was performed using Tikhonov regularization, with the hyperparameter set using cross-validation. The hyperparameter spanned $1\times {{10}^{-20}}$ to 1, in 2000 logarithmically spaced steps, and the cross validation was ten-fold using 10% of the data for training (Lionheart 2004).

Following inversion, a region of interest (ROI), $9.25\times 6.4$ mm, centered upon the electrode array’s location, was identified, as to consider the entirety of the mesh was unnecessary as sensitivity drops exponentially with increased distance from a planar array (Mueller *et al* 1999, Kao *et al* 2006), and as the physiological area of interest was restricted to the neocortex. To aid translaminar visualization, this ROI was rotated to have the layers represented normal to the brain’s surface. This was achieved by first identifying the centroids, $C$, of the overlying surface triangulation:

\begin{eqnarray*}C=\frac{1}{n+1}\underset{i=1}{\overset{n}{\sum}}\,{{\mathbf{v}}_{i}},\end{eqnarray*}

where ${{\mathbf{v}}_{i}}\ldots {{\mathbf{v}}_{n}}$ are the vectors defining the vertices and *n* is the number of dimensions. To these centroids, a plane was fitted using linear regression and the ordinary least squares solution. The normal vector of this surface was calculated and this vector’s angle in each axis identified. The FEM mesh’s vertices were then multiplied by a rotation matrix so that the surface’s normal vector only had a *z* component (i.e. so that it was directed normal to the cortical surface). Voxels deeper than 2.1 mm, a reasonable expected lower bound for layer VI (Zilles 1985, Dykes and Lamour 1988), were then removed. This $9.25\times 6.4\times 2.1$ mm ROI was linearly interpolated on a 3D grid whose lattice points were spaced 25 *μ*m apart in each axis. The smallest structure of interest was a column of approximately 300–500 m in diameter (Woolsey and Van der Loos 1970), and so 25 *μ*m was chosen to ensure structures of interest would not be a affected by interpolation, but also as a trade-off between computational overhead and down-sampling.

Image smoothing was undertaken with a Gaussian kernel with an effective full width at half maximum (FWHM) of 150 *µ*m. This FWHM was based upon the approximate thickness of the thinnest neocortical layer (Zilles 1985, Dykes and Lamour 1988), but also fulfilled the requirement of RFT that a kernel is required to be at least twice the voxel size. The data were filtered along each axis, both forwards and backwards, so as to be zero-phase filtered. Lastly, image scaling was applied to correct for the loss of power following regularization in solving the inverse problem, and was achieved by essentially applying proportional scaling. The first step was to find the time point at which a recording’s image set was maximal above zero. The mean of above half maximum changes at this peak time point was used to determine the scaling factor by finding its ratio to one. Having scaled the images, they were then converted from the MATLAB file format to NIfTI-1 file format. Realignment of images within SPM was not required as all images were reconstructed in the same FEM mesh and the same location within this mesh taken as the ROI; the ROI volume spanned all imaging areas following the post-hoc electrode alignment (appendix).

### 2.3. Parametric and non-parametric analysis

This image processing produced 21 images of $291\times 349\times 121$ voxels spanning the time period 0–40 ms for each of the 8 rats. Although it theoretically would have been possible to account for this 4th dimension of time, parametrically or non-parametrically, we opted, for clarity, to make independent statistical tests over different time bins and then pool the resulting metrics (like number of significant voxels). In order to correct for these independent tests, we made a basic Bonferroni correction to control family wise error over time bins for both approaches. That is, given the effective temporal resolution of 8 ms and 21 time bins separated by 2 ms, correction was undertaken for 5 independent tests; therefore, the significance level for any individual time bin was set to $\alpha =0.01$. These temporally corrected *p*-values are hereafter denoted by the subscript $\text{tcorr}$. In order to compare between parametric and non-parametric approaches, we ascertained the significance thresholds and the total significant image volume for the two distinct approaches over time. We also analyzed the 2 control recordings (based on post-mortem recordings in rats) using the same methods—the rationale was to consider control of the false positive rate where no experimental effect was expected.

Below a description of the employed methodology for both parametric and non-parametric analyses is given, however, a detailed didactic description of the use of the employed software has been omitted. An excellent explanation of the software, its use, and tutorials can be found at www.fil.ion.ucl.ac.uk/spm/.

#### 2.3.1. Parametric analysis.

Second level-analysis was performed, using SPM8 (www.fil.ion.ucl.ac.uk/spm/). Separately, at each of the 21 time bins, the 22 image volumes were input into a one-sample *t*-test (assuming independence) with implicit masking. The resultant design matrix was so that the estimated parameter at each voxel was the mean of that voxel over all the recordings, and so the null hypothesis was that this was equal to zero. Family wise significance was set at *p*  <  0.01 based on the correction for multiple time-bins outlined above, so that images were assessed for significant activations above zero.

#### 2.3.2. Non-parametric analysis.

These same EIT difference images were also assessed using SnPM analysis. Second-level, Monte-Carlo permutation testing was undertaken, using a SnPM toolbox (www.go.warwick.ac.uk/tenichols/software/snpm) written for the SPM software package. Separately, at each time bin, the image volumes were input into a one-sample *t*-test, without variance smoothing, but with implicit masking. The *t*-tests were with $p_{\text{tcorr}}^{\text{FWE}}$-values and so again the significance level for any single time-bin was set to *p*  <  0.01. A randomly selected subset of 1000 permutations was calculated out of the total set of possible permutations. The total number of possible permutations for 22 recordings would have totaled 4194 304 and so a Monte Carlo test was performed, so that for the chosen subset size in this study the test was for $\alpha =0.01\pm 0.0063$. This was deemed acceptable because to reduce the error to 10% would have required 37 500 permutations.

## 3. Results

### 3.1. Statistical parametric mapping

**Figure 1.**
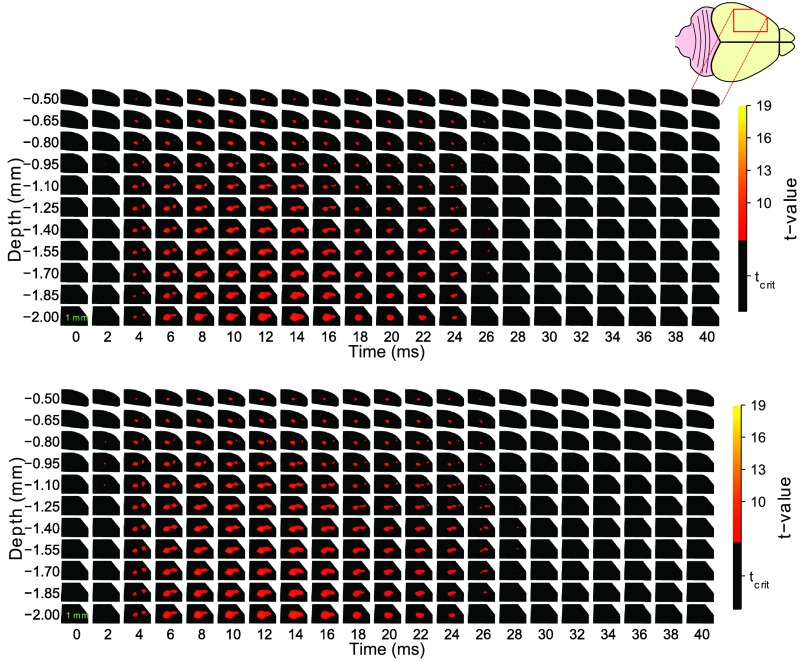
Parametric and non-parametric one-sample *t*-test results. (a) Parametric one-sample *t*-test SPMs of 22 images, $p_{\text{tcorr}}^{\text{FWE}}=0.05$. Non-significant changes are black. Each subimage is for a given time point (columns) and depth (rows). Subplots are posterior-anterior and medial-lateral in *x* and *y* axes, respectively. The significant response predominated in deeper layers and persisted in these output layers, with minimal significance in layer I (layer I 0–200 *μ*m; layer II/III 200–750 *μ*m; layer IV 750–980 *μ*m; layer V 980–1350 *μ*m; layer VI 1350–2000 *μ*m (Krieg 1946, Zilles 1985, Dykes and Lamour 1988)). (b) Non-parametric one-sample *t*-test SnPMs of 22 images, $p_{\text{tcorr}}^{\text{FWE}}=0.05$. Non-significant changes are black. The non-parametric thresholding was less conservative than the parametric, with the same location/shape of significance but a larger volume.

Physiologically plausible significant (corrected over time and volume) conductivity changes occurred in somatosensory cortex, predominantly in the period beginning at 4 ms and ending 28 ms post-stimulus (Figure 1(a)). There were also spatially small (<1300 voxels in 9M), but significant changes at all other time bins except 0 (the time of stimulation), 34, 36 and 38 ms. The image volume showed significant changes spanning the entire neocortex with the largest volume of significance (7.53 mm^3^) at 14 ms, at a depth of approximately 1.55–2 mm, which spans layers V and VI (Krieg 1946, Zilles 1985, Dykes and Lamour 1988), which are predominantly the output layers of the neocortex (Zhang and Deschenes 1997, Killackey and Sherman 2003). In addition to this, the volume of significance tapered towards the pial surface and layer I, within which, only later, feedback activity from S2 is expected (Cauller 1995). Prior to this (4–6 ms), the volume consisted of two smaller cylindrical shapes, while following this period (after 18 ms) the volume of significance reduced into a smaller more cylindrical shape, consistent with orthogonally oriented neocortical columns (Mountcastle 1997).

In the SPMs of the two post-mortem control recordings, there were no significant positive changes using the same methods.

### 3.2. Statistical non-parametric mapping

Using non-parametric methods, we found qualitatively very similar significant conductivity changes (figure 1(b)) to those observed in the parametric case (figure 1(a)). As with the parametric analysis, there were no significant positive changes at 0 ms in the non-parametric case. However, while the parametric (RFT) significant positive changes were not present in many of the later images (30–40 ms), in the non-parametric statistical maps, there were significant positive changes in all time bins except 0 ms, although as with the SPMs, these changes were predominantly within a window of 4–24 ms.

**Figure 2.**
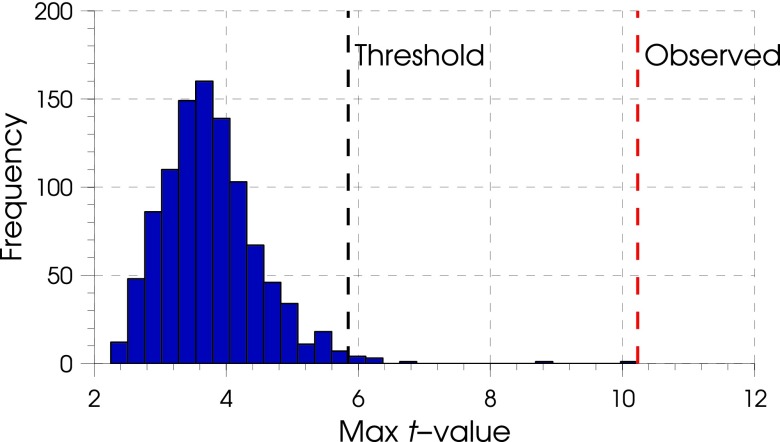
Permutation distribution of maxima *t*-statistic values for the images at 14 ms. The black dashed lines indicate the *t*-threshold for FWE corrected significance level *p*  <  0.01 based on the permuted null distribution. The observed (unpermuted) maximum *t*-value is shown by the red-dashed line and is therefore very unlikely to have occurred by chance.

The non-parametric permutation distribution of image maxima was centered within a range of *t*-values from 3 to 5 (as an example, figure 2 shows the distribution at 14 ms).

**Figure 3.**
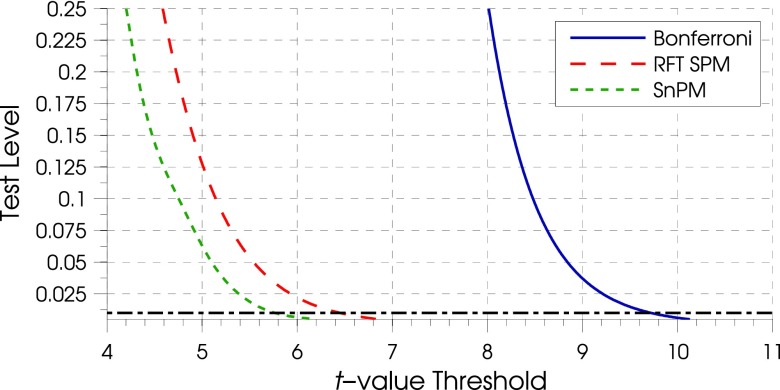
Family wise corrected error rate as a function of Bonferroni (——), RFT (— — —) and SnPM (– – –) *t*-statistical thresholds. The horizontal line indicates $\alpha =0.01$ (— · —). Note that the Bonferroni is the most conservative approach. Note also that although RFT is based on the properties (smoothness, volume) of the voxel grid, while SnPM is based on the data they have very similar *t*-value thresholds (with RFT being marginally more conservative).

As an example, the thresholds calculated with SnPM were compared to those for RFT employed in SPM, both for the images at 14 ms, and these were compared to a volumetric Bonferroni correction. This indicated that, for a given test significance level, the Bonferroni correction was extremely conservative for these EIT data. While the RFT thresholds were less conservative than the Bonferroni values, they were still less sensitive than the SnPM thresholds for an equivalent test level (figure 3).

### 3.3. Parametric and non-parametric comparison

**Figure 4.**
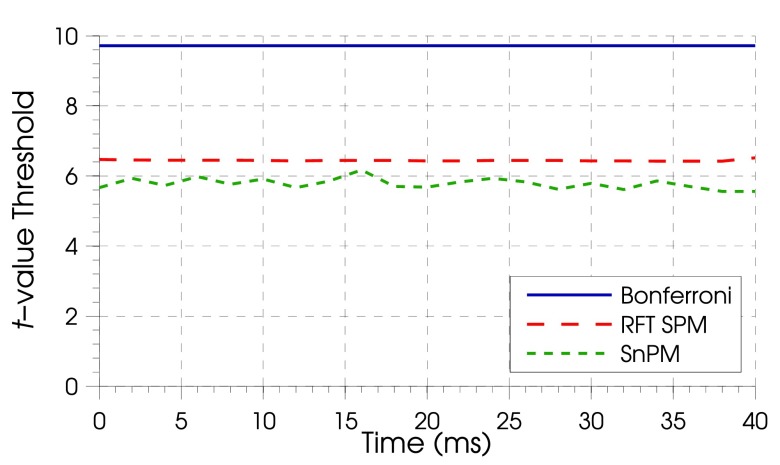
Critical *t*-thresholds for parametric and non-parametric FWE-correction methods. Bonferroni volumetric correction (——) was constant over time bins, while SPM with RFT (— — —), and SnPM (– – –) had minimal variability over time bins.

As we estimated thresholds for each time-bin independently, we were able to examine the stability of these threshold estimates for the different methods over time (figure 4). The Bonferroni thresholds were constant over the time bins, which was to be expected as they are data independent. There was only minimal variability over the time bins in the *t*-threshold with RFT SPM, with all values being within 0.08 of the mean of 6.44, suggesting the spatial smoothness of the residuals was similar in all time bins. Similarly, the non-parametrically derived *t*-threshold also remained relatively stable (mean 5.78  ±  standard deviation 0.15).

**Figure 5.**
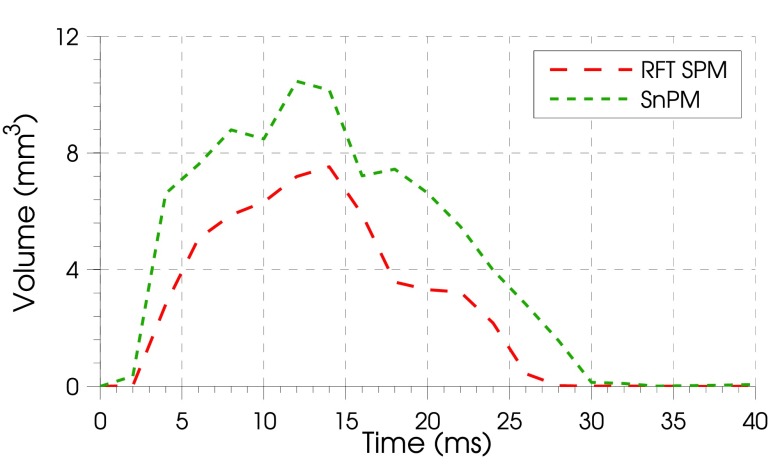
Significant volume within images at each time bin for SPM with RFT (— — —) and SnPM (– – –). The volume of significance for techniques varied similarly over time, but SPM has a smaller volume of significance at all time bins indicative of the method being more conservative.

Based on these thresholds, the total cortical volume of significant conductivity change over time for RFT SPM and the SnPM images was calculated (figure 5). As expected, a larger volume of significantly active cortex is apparent, based on the non-parametric rather than the parametric control over false positive rate.

**Figure 6.**
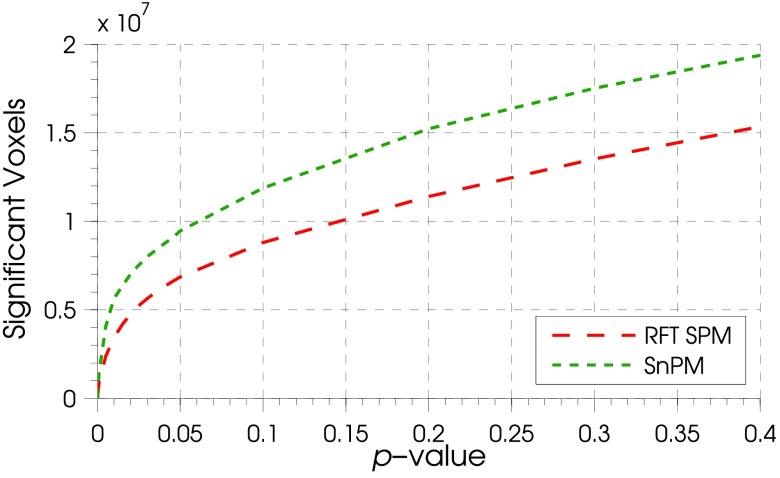
Total number of significant voxels across all times points for different FWE controlled *p*-values. The techniques had a similar relationship between *p*-value and significant voxels, but SPM with RFT (— — —) had fewer significant voxels for given p-values than for SnPM (– – –).

Over the entire time series, this same pattern was evident with the total number of significant voxels across all time points varying for different *p*-values in a similar fashion for parametric and non-parametric approaches (figure 6).

## 4. Discussion

### 4.1. Validity of SPM results and comparison to SnPM

Typically, non-parametric tests form the gold standard through which parametric tests are evaluated, due to their robustness and lack of assumptions (Nichols and Hayasaka 2003, Nichols 2012). It is for this reason the applicability of parametric testing was empirically tested, by comparison to permutation testing which lacks the assumptions of RFT, such as Gaussian error distribution and the absence of non-stationarity. Nonetheless, it is desirable to show the validity of parametric testing as the use of the parametric inference scheme opens up a very powerful and standard GLM analysis framework in which it is simple to work with complex factorial designs, including based on multiple regressors and contrasts. Although these more complex designs are tractable with non-parametric methods (Suckling and Bullmore 2004), they become less intuitive.

It was reassuring that the parametric tests were mildly more conservative than the non-parametric. The non-parametric approach has minimal assumptions and therefore we expect good control of false positive rate. It was desirable that RFT was on the conservative side of this threshold. This echoes findings reported for MEG, PET and MRI (Nichols and Holmes 2002, Singh *et al* 2003). At the outset of this work it was not clear if parametric and non-parametric testing of EIT images of fast neural activity would yield such comparable results and this underpins one of the key purposes of this study. In other words multiple testing is a serious concern for brain imaging and is something that needs addressing almost in a per-case basis, as different imaging types can have different properties that may make a generic, catch-all approach likely to fail.

We found no significant changes in either of the two dead rat controls using either method. This is encouraging as it shows the error rate is properly controlled. In an ideal world, had we had 20 dead rats, however, we would have expected one false positive at this rate (*p*  <  0.05).

The onset of spatially large significant activity in the images was at 4 ms, which, while earlier than most reports of the onset latency of forepaw somatosensory evoked potentials, is similar to the onset latency of EP activity of $3.7\pm 0.2$ ms reported by Jellema *et al* (2004). We did however observe significant small volume activations that occurred before 4 ms and after 26 to 28 ms in both image sets. First, we have controlled the false positive rate (and not eliminated it) and so these activations could well be false positives. All these regions were all  <200 *μ*m^3^ and so might also be attributed to high spatial frequency artifacts. Alternatively, it is possible that these significant peaks derive from conductivity change at other time points and are artifacts of the finite bandpass filtering used for the voltage demodulation. Given that the parametric analysis of the control recordings yielded no significant changes (but with the same EIT set up), we think that the latter explanation is most likely the key contributor to this finding, but further work would be required to confirm this.

### 4.2. Processing and interpreting SPM analysis of EIT images of fast neural activity

In order to undertake SPM analysis of EIT images, several pre-processing steps were required. Due to the absence of anatomical images or landmarks, image alignment had to be achieved through post-hoc analysis of the topography of simultaneously recorded EPs. In addition to this some pre-processing steps were essential due to the use of a planar array; the sensitive imaging volume was considerably smaller than the entire rat brain, resulting in analysis being of a ROI around the electrode array. Similarly, this ROI was rotated to visualize changes with respect to the neocortical laminar, which is different to the ‘glass brain’ projection typically used to visualize SPM results.

The interpretation of the EIT images considered in this study had multiple unique aspects, in comparison to the more common applications of SPM. Despite images being of a ROI, the high density of the electrode array yielded a considerable multiple testing problem as the number of voxels per image was 6 254 041. While it is clear that for so many voxels uncorrected testing would present a substantial risk of false positives (Bennett *et al* 2011), it also seemed likely that the poor spatial resolution and high point spread function of EIT would make interpretation of SPMs difficult. It is also worth noting that the activity measured using EIT and resulting in the SPM results are related to electrical activity rather than a secondary metabolic correlate, and therefore are more akin to the findings of EEG/MEG, than fMRI or PET results. Therefore, EIT activation maps might be more directly related to the actual substrate of brain activity: neuronal depolarization. However, further work is required to more fully understand the components of neural activity that result in the EIT signal and how much each contributes to the resultant reconstruction.

### 4.3. Study limitations

The meshes were neither specific nor scaled for each rat’s anatomy; however, the effect of this is presently not completely clear. There were also uncertainties regarding the optimal inversion approach in imaging fast neural changes with EIT using a planar array. The choice of Tikhonov regularization was one based upon its reported efficacy with planar array imaging (Mueller *et al* 1999, Kao *et al* 2006), but, in all instances, the perturbation is a single continuous object, which is unlikely to be representative of the nature of the conductivity changes, which might be expected to consist of more internally diffuse changes existing in multiple spatially separate groups.

The image scaling employed in this current study was an attempt to compensate for the effect of different hyperparameter values introducing an artificial inter-recording variance. Nonetheless, this approach of proportional scaling was not rigorously validated: proportional scaling did not result in the control recordings reaching significance, but it does limit the ability to analyze the amplitude of images’ conductivity changes.

Further examination of the appropriateness of variance smoothing in EIT images of fast neural activity, or suprathreshold cluster analysis may be required. These approaches may be essential for the technique to be applied to data with lower degrees of freedom than the cohort considered in the present study. A few simplifying assumptions were employed in the SPM analysis undertaken in this study, such as the assumption all data were independent. These assumptions were made predominantly as this was a preliminary study and also because the wealth of knowledge related to the application of SPM that exists for fMRI and PET is absent with respect to its use with EIT images. The group-level analysis of EIT images undertaken in this study was limited to fixed-effects analysis; the input to SPM was EIT conductivity change images rather than the output of first-level analysis of SPM. This was due to the lack of a canonical response function existing for the fast neural activity measured with EIT. While canonical response functions do exist for evoked response, this limitation is related to an incomplete understanding of the contributors related to the signals reconstructed with EIT.

## 5. Conclusion

In this study, it has been shown that both parametric and nonparametric testing can be used to assess the statistical significance of group EIT images of fast neural activity. Application of second-level analysis to the active experimental recordings yielded SPMs with a highly significant large volume at all post-stimulus time points from 4 to 24 ms, while for the control recordings there were no significant changes. This study represents the first attempt at validation of applied random field theory for an EIT imaging set of fast neural activity, through comparison with a permutation approach and the use of controls. In addition, this is the first study to make use of SPM second-level analysis on EIT imaging data, which has yielded some encouraging results. This validation, through comparison with non-parametric analysis, confirms that SPM can be employed, both in its current form and the full and powerful array of parametric inference schemes would in future be available to the analysis of EIT imaging of fast neural activity.

Future studies could profit from investigating the effect of varying the EIT image processing steps that, for PET and fMRI, have been determined to substantially affect the results of SPM. Similarly, examination of the efficacy of employing different smoothing methods might inform the future use of SPM on EIT image sets (Maisog and Chmielowska 1998, Skudlarski *et al* 1999). In addition, the application of SnPM might be further explored, particularly its use with smaller data sets and the use of pseudo-*t*-tests. Additionally, there are general aspects of EIT imaging of fast neural activity, especially with a planar array, that might be further explored, and such modification might be expected to affect subsequent SPM analyses.

## Appendix Electrode alignment

Electrodes for a given rat were placed on the mesh in the forward model in such a way as to minimize the difference between its EPs’ topography and that of an exemplar topography chosen from the recorded EPs. The exemplar EP was chosen by finding the recording with an EP center, $\mathbf{c}$, closest to that of the center of the electrode array. For a given EP its center, $\mathbf{c}$, was defined as:

\begin{eqnarray*}\mathbf{c}=\frac{{\sum\limits_{j=1}^{J}}\,\left({\sum\limits_{i=1}^{I}}\,E{{P}_{ji}}\mathbf{p}\right)}{{\sum\limits_{j=1}^{J}}\,{\sum\limits_{i=1}^{I}}\,E{{P}_{ji}}},\end{eqnarray*}

where $E{{P}_{ji}}\ldots E{{P}_{JI}}$, are a given recording’s EP values for the *j*th electrode and the *i*th time point, and $\mathbf{p}$ is the the electrode position. The exemplar EP was then chosen as the recording whose center had the shortest distance from the electrode array center.

The minimization between each recording and the exemplar was accomplished by minimizing the *l*2 norm between a given EP topography and the interpolation of the exemplar topography over a series of translations and rotations of the electrode positions. Translation was over a distance which spanned twice the area of the array (translation in each axis), in steps of 0.1 mm, while rotation was in the *xy* plane from $-{{10}^{\circ}}$ to 10°, in steps of 0.5°. Then each recording’s EP, *EP*_*ji*_, was compared to each point, *l*, of an interpolation of the exemplar EP, $\widehat{EP}$, and the point within this that yielded the least difference was calculated:

\begin{eqnarray*}{{\hat{l}}_{k}}=\arg \underset{l}{{\min}}\,\parallel E{{P}_{kji}}-{{\widehat{EP}}_{lji}}\parallel _{2}^{2},\end{eqnarray*}

where ${{\widehat{EP}}_{lji}},...,{{\widehat{EP}}_{LJI}}$ are the interpolated values at the *l*th location, and ${{\mathbf{\hat{l}}}_{k}}$ is the translation and rotation yielding the smallest difference between the measured recording’s EP and that of the exemplar EP, at the *j*th electrode and *i*th time point. The electrode array was then placed in the optimized location for solving the forward problem for the recordings from that rat.
